# Summer Drinks

**Published:** 1871-05

**Authors:** 


### SUMMER DRINKS.

THE first, the best, because the safest for laborers, invalids, the sedentary, for all classes, at all times of the day and night, is half a glass at a time, repeated in ten minutes if desired, of common cold water, at the temperature of the spring, or well, or reservoir, or cistern. Ice-water is more palatable, but it often kills.

Any drink which contains alcohol, even cider, root-beer, or domestic cordials, all, are not only not harmless, but are positively injurious, because the atom of alcohol, by using the strength of the next minute for the present, leaves the system that next minute just that much weaker than it would have been, had not that atom of alcohol been taken; this is the case, because that atom of alcohol has not one particle of nutriment, hence cannot supply the system with one atom of strength.

### ILLUSTRATION.

A man is allowed a loaf of bread every day to live upon, but if he eats on Monday the allowance of Monday and also foredraws and eats Tuesday's allowance, he has not only not the scant Tuesday's allowance for Tuesday, but no allowance at all.

The cause of thirst is, that so much of the watery part of the blood has escaped through the skin by perspiration, that the blood does not circulate properly, and does not strengthen the parts as it ought to do; hence the instinct of the system calls for an artificial supply of water, to dilute the blood, and for other purposes. Any liquid will do that in proportion to the pure water it contains; but as water is all water, it is of course the best.

If anything is added to the summer drink, it should contain some nutriment, so as to strengthen the body, as well as to dilute the blood for purposes of a more easy flow through the system, as any one knows that the thinner a fluid is, the more easily does it flow. Some of the nutritious and safe drinks are given below, especially for those who work in the sun of summer, all to be taken at the natural temperature of the shadiest spot in the locality. To any of them ice may be added, but it is a luxurious, not a beneficial ingredient, nor a safe one.

1. Buttermilk.

2. A pint of molasses to a gallon of water.

3. A lemon to half a gallon of water and a tea-cupful of molasses, or as much sugar.

4. Vinegar, sugar, and water are substitutes, but the vinegar is not a natural acid, contains free alcohol, hence is not as safe or healthful.

5. A thin gruel made of corn or oats, drank warm, is strengthening.

6. A pint of grapes, currants, or garden berries to a half gallon of water is agreeable.

Cold water applied to the head is very refreshing to harvesters. Wading in water abates thirst. Persons cast away at sea will suffer less from thirst, if the clothing is kept wringing wet with salt water. A piece of silk fitted in the hat at an equal distance from the hair and top of the hat, is a great protection to the head against sun heat; it is an absolute protection if one side is well covered with gold leaf. As there is always a space between the top of the head and crown of the hat, hatters should practicalize this idea.

Thirst is obviated to a certain extent by chewing some hard substance, as wax or a piece of India rubber, for it removes the unpleasant dryness of the mouth, which of itself sometimes prompts us to drink when we are really not thirsty, but feel uncomfortable, and don't know what we want.

Sometimes persons have nausea, or fullness, or metallic taste, or diarrhœa, or colic, after drinking water; but it is because they drink too much, more than enough to supply the perspiration, which is the water taken from the blood. Wells, to have good water, especially in flat lands, should be ten or fifteen feet deep.

If water is boiled to purify it, it should be abundantly shaken up when cooled, to mix the air with it.

